# Supplementary material for: Self-charging of identical grains in the absence of an external field
Source: Sci Rep. 2017 Jan 6;7:39996. doi: 10.1038/srep39996 (PMC5216333; doi:10.1038/srep39996)
Supplement: Supplementary Information [file srep39996-s1.pdf]

Supplemental Material: Something from nothing: self-charging of identical grains

R. Yoshimatsu,[1,*] N.A.M. Araújo,[2] G. Wurm,[3] H.J. Herrmann,[1,4] and T. Shinbrot[1,5,†]

[1]*Computational Physics for Engineering Materials, IfB, ETH Zurich, Wolfgang-Pauli-Strasse 27, 8093 Zurich, Switzerland*

[2]*Departamento de Física, Faculdade de Ciéncias, Universidade de Lisboa, P-1749-016 Lisboa, Portugal, and Centro de Física Teórica e Computacional, Universidade de Lisboa, P-1749-016 Lisboa, Portugal*

[3]*Faculty of Physics, University of Duisburg-Essen, Lotharstr. 1, D-47057 Duisburg, Germany*

[4]*Departamento de Física, Universidade Federal do Ceará, 60451-970 Fortaleza,Ceará, Brazil*

[5]*Department of Biomedical Engineering, Rutgers University, Piscataway, New Jersey, 08854, USA*

(Dated: September 2, 2016)

* ryutay@phys.ethz.ch

† shinbrot@rutgers.edu

SUPPLEMENTAL VIDEO 1

Discrete element simulation video of granular charging.

SUPPLEMENTAL VIDEO 2

Experiment video of granular charging.
